# A reproducible model of intramedullary spinal cord tumor in rats bearing RG2 cells

**DOI:** 10.18632/oncotarget.16045

**Published:** 2017-03-09

**Authors:** Yuandong Zhuang, Wei Zhao, Weiqiang Zhang, Hao Wei, Xinming Huang, Gangfeng Cai, Chaofeng Fu, Chunhua Wang, Rui Wang, Songsheng Shi, Weizhong Yang, Chunmei Chen

**Affiliations:** ^1^ Department of Neurosurgery, Affiliated Union Hospital of Fujian Medical University, Fuzhou, China; ^2^ Department of Medical Imaging, Affiliated Union Hospital of Fujian Medical University, Fuzhou, China

**Keywords:** intramedullary spinal cord glioma, animal model, RG2 glioma

## Abstract

Intramedullary spinal cord tumors (IMSCTs) are lethal diseases to many patients. The lack of adequate animal model has hampered the development of novel treatments. In the current study, a rodent intramedullary glioma model is established to study IMSCT progression. Fischer 344 rats received a intramedullary implantation of RG2 glioma cells. The neurological state of each rat was evaluated on daily basis using the Basso, Beattie and Bresnahan (BBB) scale. Rats implanted with RG2 cells developed significant hind limb paraplegia 20 days after implantation. Magnetic resonance imaging (MRI) scans after three weeks revealed significant intramedullary RG2 tumors in the rats. Forty days post implantation, rats were sacrificed for histopathological examination. Neuro-imaging and HE staining cross sections confirmed intramedullary RG2 glioma cells invading to the spinal cord. Thus, our model displayed many of the same invasive characteristics as human IMSCTs. This model should be a reliable and reproducible methodology to correlate well with the features of human IMSCT.

## INTRODUCTION

Intramedullary spinal cord tumors (IMSCTs) are mainly Intramedullary spinal cord gliomas (IMSCGs), which account for 2–4% of all CNS tumors. Yet, 20–25% of spinal cord malignancy are IMSCGs [1–3]. The prognosis of IMSCG remains poor, possibly due to following reasons: infiltrative nature, lack of distinct cleavage planes and limited treatments [2–5]. Surgery tumor resection remains to be the standard care for most intramedullary tumors [3, 6–8]. Radiotherapy and/or chemotherapy serve as emerging therapies for postoperative management of IMSCGs [1, 2].

The reported efficacy of surgery resection of IMSCGs between different medical centers varies greatly. The 10-year overall survival rate of patients with low-grade astrocytomas undergoing postoperative radiation therapy ranges from 40% to 91%. Meanwhile, 2-year overall survival rate of patients with high-grade spinal cord astrocytomas receiving postoperative radiation therapy remains zero [3–6, 9–17]. Until now, there are no reports about clearly defined chemotherapy for spinal cord gliomas.

The lack of proper animal models of spinal cord gliomas could be one reason for limited management of this disease. A qualified rodent spinal cord tumor model should possess certain characteristics of this condition. Further, in order to find effective treatments for IMSCGs, a reliable and reproducible IMSCG animal model needs to be developed.

In this study, we developed an animal model of IMSCG using RG2 cells. These cells were injected to Fischer344 rats. RG2 glioma has a highly invasive growth pattern, and has been utilized as a representative glioblastoma multiforme (GBM) model [18]. Here we found that our rodent IMSCG model is reproducible and efficient at many aspects, including functional progression, neuro-imaging and histopathological characterization.

## RESULTS

### Functional progression

**Figure 1:**
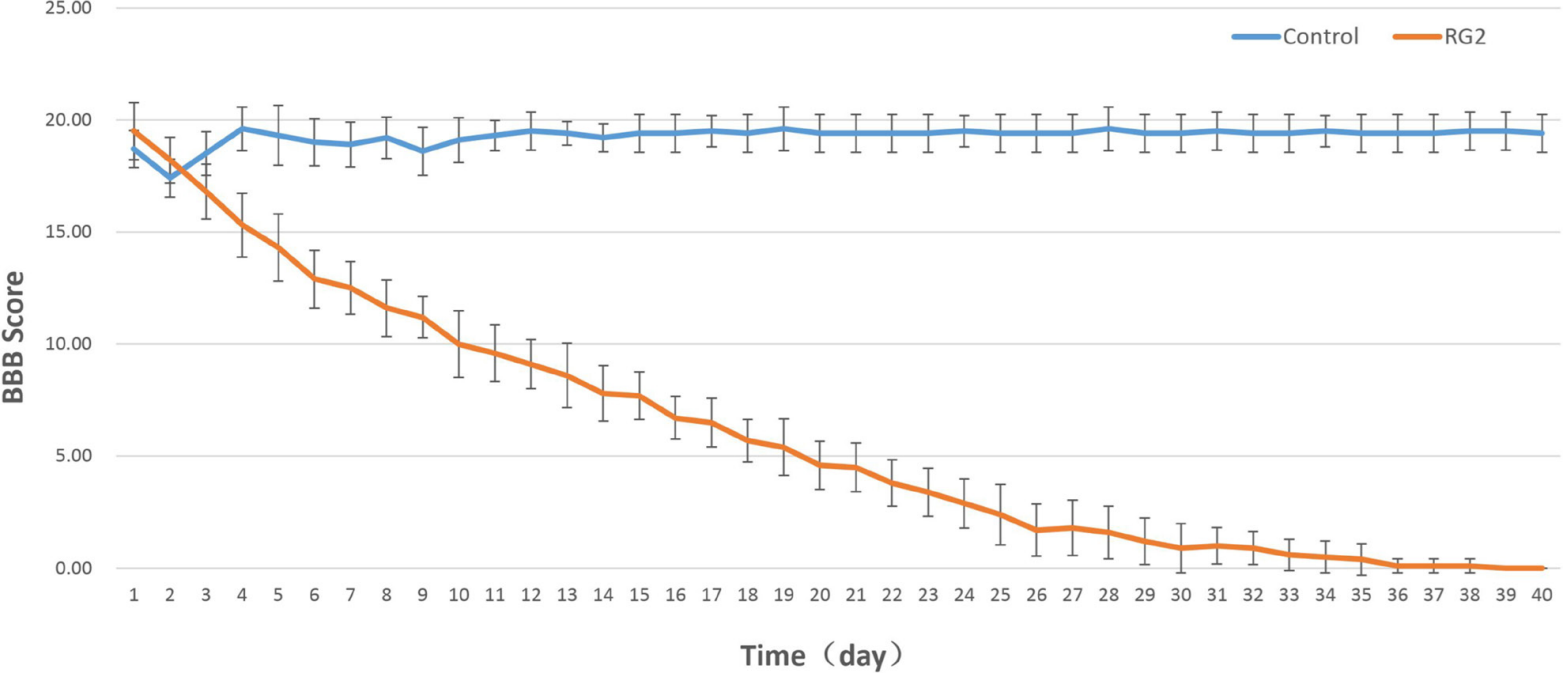
BBB score is diminished after RG2 Implantation in rats Line graph demonstrates the mean ± standard deviation (SD) of the BBB score of control vs. RG2.

Compared to control rats with sustained Basso, Bresnahan and Beattie (BBB) scores, rats implanted with RG2 cells revealed a progressive decrease in motor function at postoperative day-2. On postoperative day-14, RG2 tumor rats displayed a significantly lower BBB scores (7.8 ± 1.2) compared to rats in the control group (19.2 ± 0.6). Significant paraplegia was observed at postoperative day-20 (Figure 1). Therefore, RG2 tumor rats display lower BBB scores and paraplegia, which mimicked clinical manifestations of human IMSCT.

### Neuro-imaging

**Figure 2:**
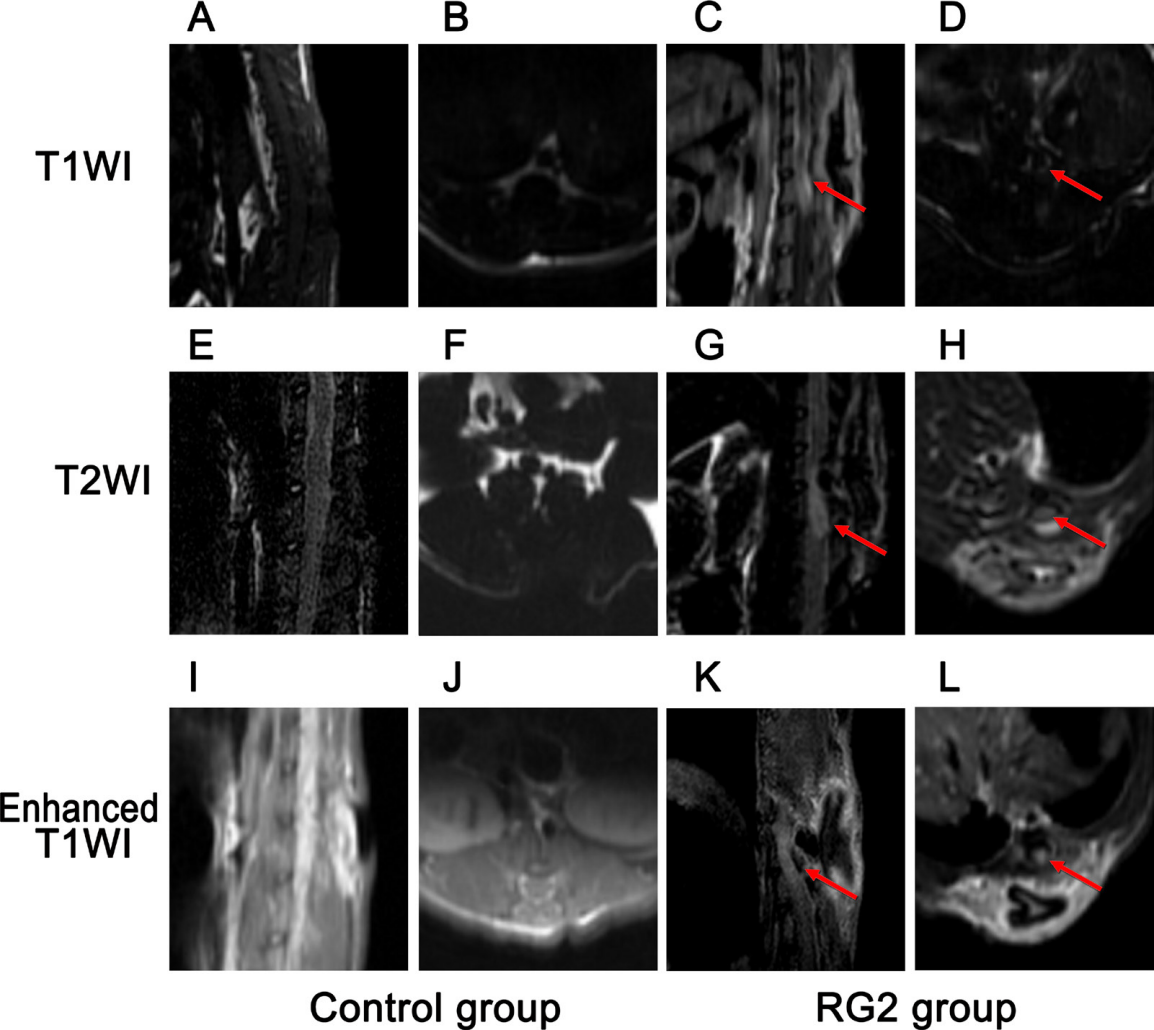
Neuro-imaging reveals phenotype in RG2 tumor rats MRI imaging of (**A**–**D**) Sagittal and axial T1WI imaging of control (left) and RG2 implantation (right), mildly hypo-intense intramedullary tumor (red arrows) in implantation group. (**E**–**H**) Sagittal and axial T2WI in control *vs*. RG2 implantation groups, mildly hyper-intense signal of intramedullary tumor (red arrows) in RG2 group. (**I**–**L**) Sagittal and axial enhanced T1WI in control *vs*. RG2 implantation group, markedly enhanced signal of intramedullary tumor (red arrows) in RG2 implantation group.

The control group revealed no evidence of intramedullary tumors in any of the magnetic resonance imaging (MRI) images acquired during the study. The RG2 tumor group presented with mildly hypo-intense signals on the T1WI intramedullary, as well as hyper-intense on the T2WI with narrowed subarachnoid spaces around tumor. The contrast enhancement of T1WI showed marked enhancement of an intramedullary lesion invading the spinal cord (at day-21, Figure 2).

### Specimens

**Figure 3:**
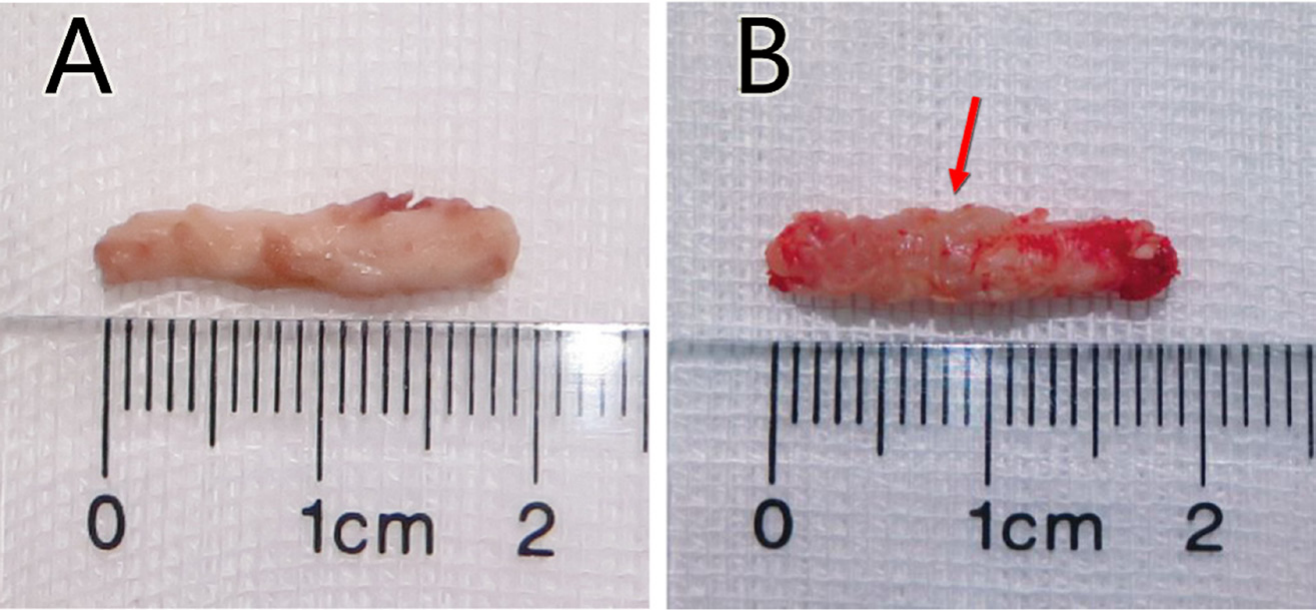
Cord specimen of the two groups (**A**) Photograph of the lower thoracic spinal cord of a control rat, no signs of tumors. (**B**) Photograph of the lower thoracic spinal cord of RG2 implantation rats presents an apparent lesion.

No evidence of any tumors was observed in the specimens of the control group. Specimens of rats injected with RG2 cells demonstrated abnormal morphology with unaided eyes and there was no clear boundary between the spinal cord and the RG2 tumors (Figure 3).

### Histopathology

**Figure 4:**
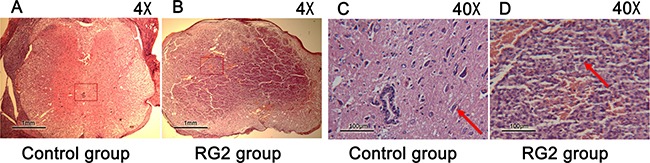
Pathological section of the two groups (**A**–**D**) Microphotographs of cross sections of spinal cords stained with HE, control rats (left), RG2 implantation rats (right). Image sizes were represented by the scale in the bottom left corners. C. Arrows indicate: normal motor neuron; D. Arrows indicate polymorphic malformed nuclei.

Histopathological examination of the control group revealed no findings of tumors in any of the cross sections. Rats implanted with RG2 cells regularly developed intramedullary spinal cord tumors. The tumors demonstrated highly cellular, well-defined lesions with invasion of surrounding gray and white matter. Within the tumors, polymorphic malformed nuclei and multinucleated cells with clearly mitotic stages were observed. Necrosis was evident by replacement with scar tissue. Endothelial proliferation was scarcely observed with limited angiogenesis (Figure 4).

## DISCUSSION

Malignant IMSCGs barely allow gross-total resection, possibly due to the absence of a cleavage plane between the tumor and normal spinal cord tissue [8, 10]. The progression of tumor recurrence inevitably leads to neurological deterioration [19–21]. Surgery remains the standard treatment for most intramedullary tumors [19, 20, 22]. This again underscores the urgent need for intramedullary tumor models that reliably mimic the invasive nature of this malignancy.

Several studies have reported growth of highly infiltrative tumors in rodents. For example, Mavinkurve *et al*. developed a model of IMSCG in rabbits which is suitable for dose escalation/toxicity studies [23]. The limited availability of monoclonal antibodies, high cost, and use of a non-glial tumor cell line (VX2 carcinoma) are all limitations of this model [23]. Caplan *et al*. revealed hind limb functional progression of rats in their model of Fischer344 rats with intramedullary implantation of 9L gliosarcoma cells and F98 glioma cells [12, 24]. Wesley Hsu *et al*., present a model of intramedullary spinal cord tumors in athymic rats using a GBM cell line obtained from a human patient [25]. The primary disadvantage of this model is the use of athymic rats, which lack a fully functional immune system.

In our study, we utilized Fischer344 rats to establish an animal model of IMSCG, via implantation of RG2 glioma cells. The RG2 glioma model exhibits similar microscopic features to the F98 glioma model, but has a highly invasive growth pattern. This is therefore a fine representative GBM model for preclinical trials [26–28]. These cells also exhibit increased expression of several oncogenes, including *Ras*, *IGF-1*, *PDGFβ*, *Erb3/HER3* and *cyclin D2* [16, 17, 29, 30]. Meanwhile, they express wild type (wt-) *p53* with a concurrent loss in the expression of the *p16/Cdkn2a/Ink4* gene locus [16, 17, 29, 30]. These cells have low levels of MHC-1 expression as compared to 9L gliomas [18, 31]. They have been utilized for numerous preclinical trials to evaluate changes in vascular permeability, disruption of the BBB, anti-angiogenic therapy, gene therapy, chemotherapy and radionuclide therapy [32–37].

In the study, the injection site was distinct from the previous IMSCG models utilizing the T5 and mid-thoracic region [24, 25]. We found that animals implanted with RG2 cells demonstrated declined hind limb motor function in a highly reproducible manner. MRI images revealed that invasion of the highly aggressive RG2 in the intramedullary region may be responsible for lower limb paralysis.

Histology studies revealed the formation of high-grade gliomas that mimic observations in humans. Furthermore, we found that rats implanted with RG2 cells had a median onset of hind limb paraplegia at day 20 ± 1.93. The progression of hind limb paraplegia in RG2 tumor rats mimics functional progression in IMSCG patients. Contrarily, the rapid proliferation of 9L and F98 cells *in vivo* may not accurately simulate the pace of tumor growth and subsequent functional deficits in patients with spinal cord gliomas.

In this rat model, MRI images definitely verified the presence and progression of tumor. The neuron-imaging characterization of this rat model parallels the features presented in humans [38]. Histopathological examination of RG2 tumor rats revealed IMSCG with an infiltrative nature and invasion of surrounding spinal cord tissue. The pathological behaviors exhibited by RG2 gliomas confirmed our MRI findings.

Recently, the RG2 glioma has been stably transfected with human Herpes virus and has been utilized to research the therapeutic efficacy of oncolytic Herpes simplex virus-1 treatment [39]. However, it has not been clear if these cells were immunogenic. Therefore, this must be considered when using the RG2 cells for immunotherapy studies. This is a potential limit of this rat model.

## MATERIALS AND METHODS

### Experimental design

Twenty (20) male Fischer 344 rats were randomized into two experimental groups receiving either intramedullary implantation of RG2 glioma cells or complete Dulbecco's modified Eagle medium (DMEM). The neurological state of each rat was evaluated daily using the Basso, Beattie and Bresnahan (BBB) scale. Magnetic resonance imaging (MRI) was conducted for detection of tumors preoperatively and 3 weeks postoperatively. Rats were sacrificed for histopathological examination 40 days postoperatively [40].

### Animals

Twenty (20) male Fischer344 rats (weight 170–200 g) were obtained from Shanghai SLAC Laboratory Animal Co. (Shanghai, China). All rats were housed in a standard facility and given free access to water and food. Rats were maintained under the following conditions: 12-hour dark/12-hour light cycle, 24 ± 2°C temperatures and 50 ± 10% humidity. The clinical signs of mice were recorded on daily bases, and if the criteria of humane endpoints were met, animals were sacrificed. Humane endpoints were considered as rapid weight loss (> 15%), abnormal changes in behavior and motion (social and eating behavior) or severe skin problems (wounds or signs of inflammation). If animals reached these endpoints, they were euthanized by exsanguination. All injections in this study were performed via anesthesia. All experimental protocols were approved by the Institutional Animal Care and Use Committee (IACUC) and Ethics Review Board (ERB) of Experimental Animal Center of Fujian Medical University (Fujian, China).

### Tumor cell line

The RG2 glioma was obtained from American Type Culture Collection (ATCC). Cell lines were incubated in DMEM (Hyclone corporation, Shanghai, China) containing 10% fetal bovine serum and 1% penicillin/streptomycin in 5% CO_2_ at 37°C [41, 42]. Tumor cells were collected and implanted into the spinal cord during the phase of exponential growth. RG2 cells were subjected to mycoplasma and microbial contamination examination every two months. Population doubling time, colony forming efficiency, and morphology were also examined routinely [43].

### Surgical technique

The experimental rats were anesthetized by intraperitoneal injection of 10% chloral hydrate solution at a dose of 1 mL/250 g body weight. Rats were placed in prone positions, in a sterile area, and their backs were shaved and disinfected with betadine solution. The spinous processes of T11 were distinguished, and a 1.5 cm midline skin incision above the spinal process was performed. A blunt dissection of the paraspinal muscle was made bilaterally to expose the spinous process, which was removed using a mini bone rongeur. Under a surgical microscope, a micro-syringe needle was inserted until its point completely entered the spinal cord. Then cell suspension containing 1*10^5^ RG2 glioma cells was slowly injected into the gray substance of the spinal cord over a five minute period. Rats in the control group received a 5 μL intramedullary injection of DMEM complete medium. Muscles were intermittently sutured. Skin was closed with 4–0 threads.

### Functional test

Functional test of hind limb locomotion was assessed by the Basso, Bresnahan and Beattie (BBB) scale. Rats were placed in an open testing area for 4 minutes of observation during continuous walking. The BBB scale is a 22-point scale ranging from 0 (no observable hind limb movement) to 21 (consistent plantar stepping and coordinated gait, consistent toe clearance, predominant paw position parallel throughout stance, consistent trunk stability, tail consistently up). When BBB score was equal to or less than 5, this was regarded as paraplegia. All rats were tested preoperatively and daily after the operation.

### Neuro-imaging

The RG2/Fischer 344 rats received MRI scanning before and three weeks after successful intramedullary inoculation with RG2 cells. MRI scanning position was T11-centered. Rats were anesthetized as previously mentioned, placed in a supine position to avoid the influence of breath. MRI images were acquired using a 3.0 T clinical unit and mouse dedicated coils. Following sagittal, axial T1WI and T2WI scanning, enhanced T1WI images were acquired by intraperitoneal injection of Gadolinium-DTPA complex (Gd-DTPA) (1 mL/250 g body weight).

### Histopathological analysis

Rats were sacrificed by overdose of 10% chloral hydrate when BBB score was equal to 5 (slight movement of 2 joints and extensive movement of the third). All surviving animals were sacrificed on day-40. The lumbar spinal cord was removed from the spinal canal. The spinal cord tumor was determined by the position of intramedullary injection. It was photographed, fixed in 4% paraformaldehyde, embedded in paraffin and sectioned at 5 μm for HE staining [43, 44].

### Statistical analysis

The BBB score was presented as mean ± standard deviation (SD). An analysis of variance (ANOVA) was applied to compare the mean BBB score between the two groups. Statistical analysis was done on SPSS 19.0 (Chicago, CA), a *p*-value less than 0.05 was considered as statistically significant.

## CONCLUSIONS

In brief, our model of IMSCT provides reliable and reproducible methodology, which correlates well with the features of human IMSCT.
